# Reliability and validity of the weight status and dietary intake measures in the COMPASS questionnaire: are the self-reported measures of body mass index (BMI) and Canada’s food guide servings robust?

**DOI:** 10.1186/1479-5868-10-42

**Published:** 2013-04-05

**Authors:** Scott T Leatherdale, Rachel E Laxer

**Affiliations:** 1 School of Public Health and Health Systems, University of Waterloo, 200 University Avenue, Waterloo, ON N2L 3G1, Canada

**Keywords:** Reliability, Validity, Body mass index, Diet, Food groups, Youth

## Abstract

**Background:**

The COMPASS study is designed to follow a cohort of ~30,000 grade 9 to 12 students attending ~60 secondary schools for four years to understand how changes in school characteristics (policies, programs, built environment) are associated with changes in youth health behaviours. Since the student-level questionnaire for COMPASS (C_q_) is designed to facilitate multiple large-scale school-based data collections using passive consent procedures, the C_q_ is only comprised of self-reported measures. The present study assesses the 1-week (1wk) test-retest reliability and the concurrent validity of the C_q_ measures for weight status and dietary intake.

**Methods:**

Validation study data were collected from 178 grade 9 students in Ontario (Canada). At time 1 (T_1_), participants completed the C_q_ and daily recoding of their dietary intake using the web-based eaTracker tool. After one week, (T_2_), students completed the C_q_ again, participants submitted their daily eaTracker logs and staff measured their height and weight. Test-retest reliability of the self-reported (SR) weight status and dietary intake measures at T_1_ and T_2_, and the concurrent validity of the objectively measured and SR weight status and dietary intake measures at T_2_ were examined using intraclass correlation coefficients (ICC).

**Results:**

Test-retest reliability for SR height (ICC 0.96), weight (ICC 0.99), and BMI (ICC 0.95) are considered substantial. The concurrent validity for SR height (ICC 0.88), weight (ICC 0.95), and BMI (ICC 0.84) are also considered substantial. The test-retest reliability for SR dietary intake for fruits and vegetables (ICC 0.68) and milk and alternatives (ICC 0.69) are considered moderate, whereas meat and alternatives (ICC 0.41), and grain products (ICC 0.56) are considered fair. The concurrent validity for SR dietary intake identified that fruits and vegetables (ICC 0.53), milk and alternatives (ICC 0.60), and grain products (ICC 0.41) are considered fair, whereas meat and alternatives (ICC 0.34) was considered slight.

**Conclusions:**

While the test-retest reliability of the measures used in this study were all high, the concurrent validity of the measures was considered acceptable. The results support the use of the self-reported COMPASS weight status and dietary intake measures for use in research where objective measures are not possible.

## Background

Age-related increases in obesity and unhealthy eating that occur among youth are cause for concern as they are associated with increased risk of cardiovascular disease, cancer and diabetes [1-4]. Since overweight, obesity, and poor eating behaviours are prevalent among youth populations [5-7], it is important to promote healthier body weights and eating habits among youth populations.

Excessive weight gain among youth is an ongoing public health problem in Canada. For instance, data from the 2007–09 Canadian Health Measures Survey (CHMS) suggest that among Canadian youth aged 15 to 19, 31% of boys and 26% of girls are overweight or obese [6]. This represents a dramatic population-level increase from 25 years ago where only 14% of boys and 14% of girls were considered overweight or obese [6]. According to the World Health Organization (WHO), adequate fruit and vegetable intake is the most important dietary indicator related to weight management and disease prevention [8]. For optimal health outcomes, the Canada Food Guide makes recommendations for teens aged 14 to 18 years pertaining to daily recommendations for the consumption of fruits and vegetable, grain products, milk and alternatives, and meats and alternatives [9]. However, according to the 2010 Canadian Community Health Survey (CCHS), only 48% of males and 50% of females 12 to 19 years of age consumed the recommended five daily servings of fruits and vegetables [10]. Although these national recommendations exist, data pertaining to the prevalence of youth meeting these benchmarks for grain products, milk and alternatives, and meats and alternatives are not available in the published literature. A simple tool to measure whether youth are consuming the appropriate number of servings per day for the four food groups could provide valuable insight for stakeholders developing programs or policies to promote healthy eating behaviours.

Since youth spend a large part of their days at school, schools are increasingly tasked with preventing overweight and obesity and promoting healthy eating behaviour among youth populations. However, school stakeholders are not provided with the tools or resources necessary to develop evidence-based programs related to overweight, obesity, and healthy eating [11,12]. The COMPASS study was designed to fill this gap [http://www.compass.uwaterloo.ca]; it is a longitudinal study (starting in 2012–13) following a cohort of ~30,000 grade 9 to 12 students attending ~60 Ontario secondary schools for four years to understand how changes in school environment characteristics (policies, programs, built environment) are associated with changes in youth health behaviours. COMPASS originated to provide school stakeholders with the evidence to guide and evaluate school-based interventions related to obesity and healthy eating (as well as tobacco use, alcohol and drug use, physical activity and sedentary behaviour, school connectedness, bullying, and academic achievement). The student-level questionnaire for COMPASS has been designed to facilitate multiple large-scale school-based data collections. As such, there were key issues for measuring both weight status and dietary intake that had to be considered when the questionnaire for COMPASS questionnaire was developed.

To accurately assess and monitor weight and nutritional status among youth, researchers often rely on retrospective self-reports, requiring the recall of behaviours. While accuracy may be compromised due to recall problems or social desirability bias (i.e., misreporting on sensitive or embarrassing behaviours to appear more favourable) [13], any misreporting is likely to remain consistent over time [14]. This is most important for longitudinal research and tracking youth over time (such as in the COMPASS study). While objective measures of height, weight, and dietary behaviours provide the most accurate and valid results, they are often costly, time consuming, and not feasible for use in large population-based studies [13]. For example, objective measures of height and weight require active consent procedures and the time of a trained researcher, while those for dietary behaviours require costly machinery and may be considered invasive and impractical in non-clinical settings (i.e., doubly labeled water), or are far too tedious and outside the realm for classroom based studies (i.e., 7-day food recall). It is therefore important to develop valid tools to measure self-reported weight status and dietary behaviours (i.e., surveys, questionnaires) that offer the advantage of being quick, inexpensive, and easy to administer in large samples using passive consent procedures.

The protocol for COMPASS involves active information with passive consent procedures. This ensures representative whole-school samples to inform and evaluate program and policy decisions at the school-level. To facilitate this large-scale data collection, enable fast and accurate processing of questionnaires, and minimize labour costs and transcription errors, the student-level questionnaire needed to be in a machine-readable format. The use of passive consent and questionnaire processing protocols do not allow for objective measures of height and weight of whole school samples. This is consistent with previous large scale studies using similar methods [11,15,16]. As such, we needed to develop and test the psychometric properties of self-reported height and weight measures to be used in COMPASS.

To minimize the burden on schools and students and ensure survey completion in one class period (~30-40 minutes), it was necessary that the questionnaire be no more than 12 pages long. This created a challenge in selecting items to balance both the depth of the core measures associated with each behavioural outcome and the breadth of data that could be measured in each domain. Within this protocol restriction, it was not possible to use a detailed food frequency questionnaire to measure eating behaviours in COMPASS (i.e., limited to 1-page for measuring dietary intake). The purpose of this study was to assess the 1-week (1wk) test-retest reliability and the concurrent validity of the self-reported COMPASS questionnaire measures used to determine weight status and dietary intake associated with Canada Food Guide servings.

## Methods

### Data collection

Validation study data were collected using a convenience sample of 178 students in grade 9 from four schools in Southwestern Ontario (Canada). Participants completed the COMPASS questionnaire (C_q_) during class time (~30 min) on two separate occasions between September and December 2011. At time 1 (T_1_), staff administered the C_q_ in classrooms using a common protocol and standardized instructions. Once the C_q_ was completed, student participants were instructed on the eaTracker food consumption diary [17]. eaTracker is a web-based dietary measurement tool developed by the Dieticians of Canada [17]; participants enter detailed information on their daily food and beverage consumption [http://www.eatracker.ca]. Participants completed daily food consumption logs using the eaTracker website for seven days following T_1_. After one week, the C_q_ was re-administered to the same students (T_2_). A self-generated code was included on the cover sheet of the C_q_ to permit accurate tracking of participants over time. Upon T_2_ completion of the C_q_ study staff verified that all participants completed their daily eaTracker logs and uploaded their data. Each participant’s weight and height were then measured by study staff consistent with existing protocols [18,19]. Students were provided an honorarium of $35 for completing the C_q_ at T_2_. Ethics approval was granted by the University of Waterloo Office of Research Ethics and participating school board and school ethics committees.

### Measures

#### Overweight and obesity

**Figure 1:**
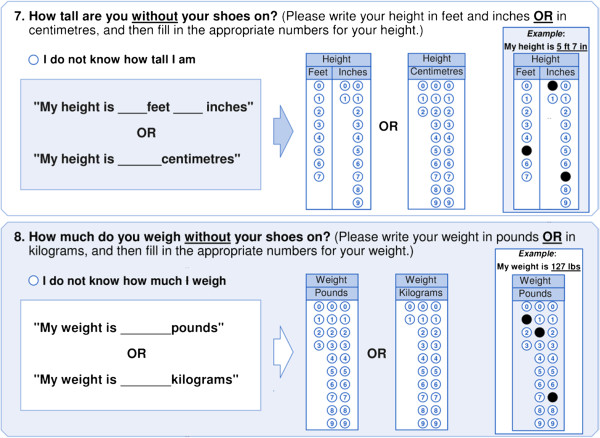
COMPASS questionnaire measures of height and weight used to calculate BMI.

Self-reported height and weight were measured using two questions in the C_q_ (see Figure 1). The self-reported height and weight items were consistent to those used in the Youth Risk Behavior Survey (YRBS) [18] and the School Health Action, Planning and Evaluation System (SHAPES) [20]. We also provided respondents with both metric and imperial response options and a blank line prefaced by “My weight is ____ pounds/kilograms” and “My height is ____ inches/centimetres”. Because of the different format of response options from the YRBS, and the preface wording from SHAPES, it was important to establish the test–retest reliability and concurrent validity of the height and weight items used in the C_q_. Objective measures of height and weight were taken by study staff. Standing height was objectively measured to the nearest 1 cm with shoes off, feet together, and back against the wall with a horizontal measuring tape. Body weight was measured to the nearest 1 kg using a digital scale with participants wearing light clothes and no shoes. Body Mass Index (BMI) was calculated for each participant using self-reported and objectively measured body weight (kg) and height (m) (BMI = kg/m^2^). Overweight and obesity status were then determined using the International Obesity Task Force BMI classification system [21] based on age and sex adjusted BMI cut-points. A participant was classified as overweight if their age and sex adjusted BMI cut-point was ≥25 and <30 kg/m^2^, and obese if their age and sex adjusted BMI cut-point was ≥30 kg/m^2^. Students with an age and sex adjusted BMI of 18 < 25 kg/m^2^ were classified as normal weight.

#### Dietary intake

**Figure 2:**
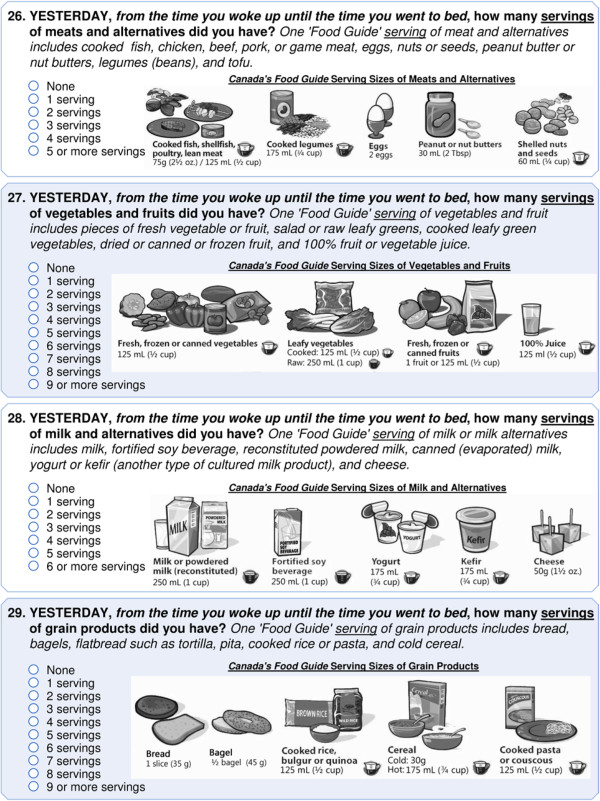
**COMPASS questionnaire eating behaviour measures.** Images from Canada’s Food Guide. Health Canada, 2011. Reproduced with the permission of the Minister of Health, 2011.

The C_q_ includes four questions to measure respondent consumption of the four food groups outlined in the Canada Food Guide [9]. Respondents were asked to report: “Yesterday, from the time you woke up until the time you went to bed, how many servings of meats and alternatives did you have? *One ‘Food Guide’ serving of meat and alternatives includes cooked fish, chicken, beef, pork, or game meat, eggs, nuts or seeds, peanut butter or nut butters, legumes (beans), and tofu*;” “Yesterday, from the time you woke up until the time you went to bed, how many servings of vegetables and fruits did you have? *One ‘Food Guide’ serving of vegetables and fruit includes pieces of fresh vegetable or fruit, salad or raw leafy greens, cooked leafy green vegetables, dried or canned or frozen fruit, and 100% fruit or vegetable juice*;” “Yesterday, from the time you woke up until the time you went to bed, how many servings of milk and alternatives did you have? *One ‘Food Guide’ serving of milk or milk alternatives includes milk, fortified soy beverage, reconstituted powdered milk, canned (evaporated) milk, yogurt or kefir (another type of cultured milk product), and cheese;*” and, “Yesterday, from the time you woke up until the time you went to bed, how many servings of grain products did you have? *One ‘Food Guide’ serving of grain products includes bread, bagels, flatbread such as tortilla, pita, cooked rice or pasta, and cold cereal.*” (see Figure 2). Health Canada granted permission for the COMPASS study to use the Canada Food Guide images for the types of servings and serving sizes for food groups measured within the C_q_. We used these self-reported measures to determine the number of servings of each food group consumed, and whether the respondents met the recommended number of servings for each food groups as outlined in the Canada Food Guide. Participants are classified as meeting the food guide recommendations based on the following minimum number of servings: meats and alternatives (2 for females, 3 for males), fruits and vegetables (7 for females, 8 for males), milk and alternatives (3 for females and males), and grain products (6 for females, 7 for males). The eaTracker online tool was used to measure daily food consumption. As calculated in eaTracker, the dietary intake scores for Day 6 were used to determine both the total number of servings for each of the four food groups, and whether or not the respondent met the thresholds for the Canada Food Guide recommendations for teens.

#### Analyses

Conventional descriptive statistics were used for the self-reported and measured weight status and dietary intake measures (examined by sex). Test-retest reliability of the self-reported weight status and dietary intake measures at T_1_ and T_2_, and the concurrent validity of the objectively measured and self-reported weight status and dietary intake measures at T_2_ were examined using intraclass correlation coefficients (ICC). Given gender biases in self-reported height and weight among youth identified in previous research [22], we also examined the concurrent validity of the weight status measures by sex. For the purpose of comparison to previous studies, test-retest reliability and concurrent validity were also determined using Cronbach’s Alpha and Spearman correlations for weight status and dietary intake (Spearman correlations were used instead of Pearson correlations since these data were not normally distributed), and weighted Kappa for dietary intake. In order to make our results meaningful and easier to interpret for a broader audience of stakeholders and researchers, correlation rating interpretations [23,24] are also provided to help with the interpretation of the strength of the results presented for our reliability and validity values: ICC (0.00 to 0.10 virtually none, 0.11 to 0.40 slight, 0.41 to 0.60 fair, 0.61 to 0.80 moderate, and 0.81 to 1.0 substantial); Spearman correlation (0.10 to 0.30 weak, 0.30 to 0.50 moderate, >0.50 strong); Cronbach’s Alpha (<0.50 unacceptable, 0.50 to 0.59 poor, 0.60 to 0.69 questionable, 0.70 to 0.79 acceptable, 0.80 to 0.89 good, ≥ 0.90 excellent); and, Kappa statistic (≤0.20 slight, 0.21 to <0.40 fair, 0.40 to <0.60 moderate, 0.60 to <0.80 substantial, 0.81 to 1.00 excellent). Means and standard deviations for the objectively measured and C_q_ T_2_ self-reported weight status and dietary intake measures were calculated to determine the difference between the self-reported and objective measures and the accuracy (over or under reporting) of the self-reported measures. The statistical package SAS 9.2 was used for all analyses.

## Results

### Descriptive statistics

Table 1 presents descriptive statistics of the sample and the weight status and dietary intake measurements. The sample was 52.8% (n = 94) female and 47.2% (n = 84) male. Overall, self-reported T_1_ and T_2_ data required to calculate BMI were only available from 139 respondents (78.1%); data were missing from 30.9% of females (n = 29) and 11.9% of males (n = 10).

**Table 1:** Means and standard deviations of COMPASS weight status and dietary intake measures

	**Males**			**Females**			**Total**		
	***N***	***μ***	**SD**	***N***	***μ***	**SD**	***N***	***μ***	**SD**
**Weight Status***(Time 1 self-reported)*									
Height (cm)	74	173.85	9.41	65	163.96	6.90	139	169.06	9.56
Weight (kg)	74	65.61	13.19	65	57.21	10.48	139	61.62	12.71
Body Mass Index (kg/m^2^)	74	21.65	3.68	65	21.19	2.95	139	21.45	3.35
**Weight Status***(Time 2 self-reported)*									
Height (cm)	74	173.97	9.29	65	163.50	7.95	139	168.90	10.02
Weight (kg)	74	65.71	13.09	65	57.55	10.75	139	61.84	12.72
Body Mass Index (kg/m^2^)	74	21.65	3.68	65	21.47	3.26	139	21.59	3.48
**Weight Status***(Time 2 measured)*									
Height (cm)	74	174.07	8.88	65	163.69	6.72	139	169.05	9.38
Weight (kg)	74	67.68	13.98	65	60.30	11.35	139	64.18	13.33
Body Mass Index (kg/m^2^)	74	22.24	3.76	65	22.42	3.44	139	22.34	3.60
**Dietary Intake***(Time 1 self-reported)*									
Meat and alternatives (0–5 servings)	84	2.50	1.07	94	1.91	0.89	178	2.19	1.02
Fruits and vegetables (0–9 servings)	84	3.42	2.03	94	3.27	2.25	178	3.34	2.15
Milk and alternatives (0–6 servings)	84	3.56	1.51	94	2.36	1.48	178	2.93	1.61
Grain products (0–9 servings)	84	3.76	1.85	94	2.64	1.31	178	3.17	1.68
**Dietary *Intake****(Time 2 self-reported)*									
Meat and alternatives (0–5 servings)	84	2.26	0.92	94	1.69	0.89	178	1.96	0.95
Fruits and vegetables (0–9 servings)	84	3.64	2.30	94	3.35	2.33	178	3.49	2.31
Milk and alternatives (0–6 servings)	84	3.24	1.63	94	1.97	0.98	178	2.57	1.46
Grain products (0–9 servings)	84	3.92	1.96	94	3.02	1.70	178	3.44	1.88
**Dietary Intake***(Time 2 measured)*									
Meat and alternatives (0–5 servings)	84	2.07	1.11	94	1.58	1.08	178	1.81	1.11
Fruits and vegetables (0–9 servings)	84	3.65	2.42	94	3.28	2.34	178	3.46	2.38
Milk and alternatives (0–6 servings)	84	2.88	1.90	94	1.71	1.26	178	2.26	1.69
Grain products (0–9 servings)	84	4.99	2.27	94	4.05	2.06	178	4.49	2.20

### Test-retest reliability

As shown in Table 2, test-retest reliability for self-reported height (ICC 0.96), weight (ICC 0.99), and BMI (ICC 0.95) are considered substantial. The test-retest reliability for self-reported dietary intake for fruits and vegetables (ICC 0.68) and milk and alternatives (ICC 0.69) are considered moderate, whereas meat and alternative (ICC 0.41), and grain products (ICC 0.56) are considered fair. Test-retest reliability for meeting the food guide recommendations identified that for fruits and vegetables (ICC 0.53) and milk and alternatives (ICC 0.54), the correlations are considered fair, whereas meat and alternative (ICC 0.27), and grain products (ICC 0.38) are considered slight. The strength of the Spearman correlations, Cronbach’s Alpha and Kappa values were consistent with the ICC estimates.

**Table 2:** Test-retest reliability of the COMPASS weight status and dietary intake measures

	***N***	**Intraclass correlation**	**Cronbach’s alpha**	**Spearman correlation**	**Kappa/Weighted Kappa**
		**ICC**	***α***	***rho***	***μ (SD)***
**Weight Status **^§^					
Height (cm)	139	0.96	0.98	0.96 (p < 0.001)	-
Weight (kg)	139	0.99	0.99	0.99 (p < 0.001)	-
Body Mass Index (kg/m^2^)	139	0.95	0.98	0.95 (p < 0.001)	-
**Dietary Intake **^§^					
Meat and alternatives (0–5 servings)	178	0.41	0.59	0.42 (p < 0.001)	0.28 (0.04)
Fruits and vegetables (0–9 servings)	178	0.68	0.81	0.69 (p < 0.001)	0.52 (0.04)
Milk and alternatives (0–6 servings)	178	0.69	0.83	0.71 (p < 0.001)	0.48 (0.04)
Grain products (0–9 servings)	178	0.56	0.73	0.57 (p < 0.001)	0.42 (0.05)
**Meeting Food Guide Recommendation **^§^					
Meat and alternatives ^a^	178	0.27	0.44	0.28 (p < 0.001)	0.27 (0.07)
Fruits and vegetables ^b^	178	0.53	0.73	0.58 (p < 0.001)	0.53 (0.12)
Milk and alternatives ^c^	178	0.54	0.71	0.55 (p < 0.001)	0.54 (0.06)
Grain products ^d^	178	0.38	0.56	0.39 (p < 0.001)	0.38 (0.12)

^§^ Self-reported measure at Time 1 and Time 2.

^a^ ≥2 servings for females, ≥3 servings for males.

^b^ ≥7 servings for females, ≥8 servings for males.

^c^ ≥3 servings for females and males.

^d^ ≥6 servings for females, ≥7 servings for males.

### Comparison between self-report and objectively measured

Table 3 demonstrates the differences for self-reported and objectively measured weight, height, and dietary intake. On average, self-reported measures for height, weight and BMI were underestimated relative to the measured values for the whole sample and when examined by sex. For instance, on average in the whole sample, weight was underestimated by 2.34 kg, height was underestimated by 1.5 cm, and therefore BMI was also underestimated (by 0.76 kg/m^2^). For dietary intake, the mean difference in self-reported consumption of grain product was negative (underestimated by 1.04 servings), whereas mean differences were positive for self-reported consumption of meat and alternatives (overestimated by 0.15 servings) and milk and alternatives (overestimated by 0.31 servings). Although the mean difference in self-reported fruits and vegetables consumption was positive, it was only overestimated by 0.03 servings. Self-reported measures of grain products underestimated the true prevalence of respondents meeting the food guide recommendation by 13%, whereas self-reported measures of meat and alternatives and milk and alternatives overestimated the prevalence of respondents meeting the food guide recommendation by 7% and 11% respectively. Self-reported fruit and vegetable intake only overestimated the prevalence of respondents meeting the food guide recommendation by 2%.

**Table 3:** Comparison between self-reported (S) and objectively measured (M) weight status and dietary intake

	***N***	**S**^**§**^	**M**	**S – M**	**#**	**#**	**#**
		***μ (SD)***	***μ (SD)***	***μ (SD)***	**Over**^**†**^	**Under**^**†**^	**Same**^**†**^
**Weight Status (males and females)**							
Height (cm)	139	168.90 (10.02)	169.05 (9.38)	−0.15 (4.81)	63	48	29
Weight (kg)	139	61.84 (12.72)	64.18 (13.33)	−2.34 (3.51)	18	110	12
Body Mass Index (kg/m^2^)	139	21.59 (3.48)	22.35 (3.60)	−0.76 (1.87)	16	82	42
**Weight Status (males only)**							
Height (cm)	74	173.97 (9.29)	174.07 (8.88)	−0.14 (5.17)	36	22	16
Weight (kg)	74	65.71 (13.09)	67.68 (13.98)	−1.96 (4.11)	12	55	7
Body Mass Index (kg/m^2^)	74	21.65 (3.68)	22.24 (3.76)	−0.60 (1.97)	11	41	22
**Weight Status (females only)**							
Height (cm)	65	163.50 (7.95)	163.69 (6.72)	−0.26 (4.46)	27	25	13
Weight (kg)	65	57.55 (10.75)	60.30 (11.35)	−2.82 (2.69)	5	55	5
Body Mass Index (kg/m^2^)	65	21.47 (3.26)	22.42 (3.44)	−1.00 (1.77)	4	41	20
**Dietary Intake**							
Meat and alternatives (0–5 servings)	178	1.96 (0.95)	1.81 (1.11)	0.15 (1.15)	61	22	95
Fruits and vegetables (0–9 servings)	178	3.49 (2.31)	3.46 (2.38)	0.03 (2.26)	55	47	76
Milk and alternatives (0–6 servings)	178	2.57 (1.46)	2.26 (1.46)	0.31 (1.37)	65	38	75
Grain products (0–9 servings)	178	3.44 (1.88)	4.49 (2.20)	−1.05 (2.12)	29	87	62
**Meeting Food Guide Recommendation**							
Meat and alternatives ^a^	178	0.47 (0.50)	0.40 (0.49)	0.07 (0.52)	30	18	130
Fruits and vegetables ^b^	178	0.10 (0.29)	0.08 (0.27)	0.02 (0.27)	8	5	165
Milk and alternatives ^c^	178	0.48 (0.50)	0.37 (0.48)	0.11 (0.48)	31	12	135
Grain products ^d^	178	0.11 (0.31)	0.24 (0.43)	−0.13 (0.41)	5	28	145

^§^ Self-reported measure at Time 2.

^†^ Values were rounded to 0 decimal places (i.e., 21.59 = 22).

^a^ ≥2 servings for females, ≥3 servings for males.

^b^ ≥7 servings for females, ≥8 servings for males.

^c^ ≥3 servings for females and males.

^d^ ≥6 servings for females, ≥7 servings for males.

### Concurrent validity

As shown in Table 4, the concurrent validity for self-reported height (ICC 0.88), weight (ICC 0.95), and BMI (ICC 0.84) are considered substantial for the entire sample. Among males, concurrent validity for self-reported height (ICC 0.84), weight (ICC 0.95), and BMI (ICC 0.85) are considered substantial. Similarly, among females, concurrent validity for self-reported height (ICC 0.82), weight (ICC 0.94), and BMI (ICC 0.83) are considered substantial. The concurrent validity for self-reported dietary intake identified that fruits and vegetables (ICC 0.53), milk and alternatives (ICC 0.60), and grain products (ICC 0.41) are considered fair, whereas meat and alternative (ICC 0.34) was considered slight. The concurrent validity for meeting the food guide recommendations identified that meats and alternatives (ICC 0.45), fruits and vegetables (ICC 0.54), and milk and alternatives (ICC 0.52) are considered fair, whereas grain products (ICC 0.36) are considered slight. The strength of the Spearman correlations, Cronbach’s Alpha and Kappa values were consistent with the ICC estimates, although the Spearman correlations for dietary intake of meats and alternatives and meeting the food guide recommendation for grain products were considered moderate.

**Table 4:** Validity of COMPASS self-reported (S) and objectively measured (M) weight status and dietary intake measures

	***N***	**Intraclass correlation**	**Cronbach’s `lpha**	**Spearman correlation**	**Kappa/Weighted Kappa**
		**ICC**	***α***	***rho***	***μ (SD)***
**Weight Status (males and females) **^§^					
Height (cm)	139	0.88	0.94	0.88 (p < 0.001)	-
Weight (kg)	139	0.95	0.98	0.96 (p < 0.001)	-
Body Mass Index (kg/m^2^)	139	0.84	0.92	0.86 (p < 0.001)	-
**Weight Status (males only) **^§^					
Height (cm)	74	0.84	0.91	0.84 (p < 0.001)	-
Weight (kg)	74	0.95	0.98	0.96 (p < 0.001)	-
Body Mass Index (kg/m^2^)	74	0.85	0.92	0.86 (p < 0.001)	-
**Weight Status (females only) **^§^					
Height (cm)	65	0.82	0.89	0.83 (p < 0.001)	-
Weight (kg)	65	0.94	0.99	0.97 (p < 0.001)	-
Body Mass Index (kg/m^2^)	65	0.83	0.93	0.86 (p < 0.001)	-
**Dietary Intake **^§^					
Meat and alternatives (0–5 servings)	178	0.34	0.56	0.38 (p < 0.001)	0.36 (0.05)
Fruits and vegetables (0–9 servings)	178	0.53	0.70	0.54 (p < 0.001)	0.47 (0.05)
Milk and alternatives (0–6 servings)	178	0.60	0.77	0.63 (p < 0.001)	0.48 (0.04)
Grain products (0–9 servings)	178	0.41	0.64	0.47 (p < 0.001)	0.33 (0.05)
**Meeting Food Guide Recommendation **^§^					
Meat and alternatives ^a^	178	0.45	0.63	0.46 (p < 0.001)	0.45 (0.07)
Fruits and vegetables ^b^	178	0.54	0.70	0.54 (p < 0.001)	0.54 (0.11)
Milk and alternatives ^c^	178	0.52	0.69	0.52 (p < 0.001)	0.51 (0.06)
Grain products ^d^	178	0.36	0.58	0.41 (p < 0.001)	0.37 (0.08)

^§^ Self-reported measure at Time 2 and objective measure taken at Time 2.

^a^ ≥2 servings for females, ≥3 servings for males.

^b^ ≥7 servings for females, ≥8 servings for males.

^c^ ≥3 servings for females and males.

^d^ ≥6 servings for females, ≥7 servings for males.

## Discussion

Large scale school-based studies aiming to improve youth health behaviours require instruments that are easily administered in large populations, simple to fill out, cost-effective, reproducible, and accurate [11,15,16,20]. However, due to the complexity, cost, and necessity of active consent procedures when objectively measuring health behaviours and weight status of youth populations, objective measures are often not feasible or appropriate. Yet, in order to evaluate school-based programs and policies associated with obesity prevention or dietary intake, researchers require measures that provide both reliable estimates over time and valid measures of the constructs they intend to change. The present study was designed to assess the test-retest reliability and concurrent validity of self-reported measures of height, weight, and dietary intake within the C_q_. We demonstrate that the concise yet simple measures in the C_q_ provide reliable and valid measures for collecting self-reported data on weight status and eating patterns for use in large scale school-based studies.

### Reliability of the C_q_ measures

We identified that the C_q_ self-report measures of weight and height (and the derived measure for BMI) were highly reliable with 1-week test-retest (Cronbach’s Alpha >0.98 for all measures). This is consistent with available evidence from studies examining both 1-week test retest [18] and 2-week test-retest [18] of self-reported height and weight. We also identified that the C_q_ self-report measures of dietary intake pertaining to the four food groups and meeting Canada’s Food Guide recommendations had sufficient 1-week test-retest reliability. This is consistent with previous research [25-28]. Given that eating patterns and food choices fluctuate day-to-day, it is to be expected that there would be some variability in self-reported dietary behaviours of youth between weeks and days and that reports would not be as robust as for weight status measures [29]. As such, a 50% agreement is considered to be reasonable and sufficient for measuring reliability of self-reported dietary intake in youth [29]. And since diets vary daily, a 7-day food record should provide estimates of population means for nutrients [30,31] and be sufficient to capture normal eating patterns in the adolescent population. No research had previously examined the reliability or validity of dietary intake measures based on the four food groups outlined in Canada’s Food Guide.

### Validity of the C_q_ measures

We identified that the concurrent validity of the C_q_ self-report measures of weight and height (and the derived measure for BMI) were substantial although discrepancies between self-reported and actual measured height and weight did exist. Consistent with the literature [18,32-35], the C_q_ measures underestimated weight (by 2.34 kg on average) and subsequently BMI (by 0.76 kg/m^2^ on average). While the average self-reported height was an underestimate of actual measured height (by only −0.15 cm), the majority of respondents (45% of the sample) actually over-report their height, a finding consistent with the literature [18,32,36,37]. Although relying exclusively on self-reported height and weight measures may provide erroneous estimates for overweight and obesity, the C_q_ derived measure for BMI was similar in robustness to other similar measures [18,20,32,34,38,39], and only underestimated BMI by 0.76 kg/m^2^. It appears that the C_q_ measures can provide valid measures of BMI for use in large-scale school-based data collections requiring self-report measures among both males and females. Moreover, since the C_q_ is designed for use in a longitudinal study of youth populations (i.e., examining temporal patterns and tracking the same youth over time), any modest biases in the data should remain consistent within students over time [14].

Since there is no gold standard to assess dietary behaviours, determining the validity of a dietary measurement technique must be done in comparison to one that would seemingly capture more accurate measures [40]. Advances in technology and the increased accessibility of the Internet allow for the use of web-based alternatives to the 24-hour recall or a demanding food-frequency questionnaire [41,42]. Among youth populations, there are benefits to using an Online tool (i.e., the eaTracker) as a gold standard relative to the traditional food-frequency questionnaires [41,42] as they offer immediate checks for incomplete responses, the opportunity to update consumption of food products at any time throughout the day, and the use of photographs to enhance portion size estimation [41]. The comparison of the C_q_ dietary intake responses to the online eaTracker data identified that the C_q_ measures of dietary intake and meeting food guide recommendations demonstrated sufficient validity for use as self-reported measures in a school-based survey tool.

It is difficult to compare the findings in this study to the literature because no other studies have used Canada’s Food Guide (or their international equivalent) to measure dietary behaviours based on food group guidelines. Most validation studies, rather, have made comparisons of eating behaviours in youth to servings as recommended in Canada’s Food Guide or the American Food Pyramid [25,43], but none have used Canada’s Food Guide itself as the unit of measure. Studies have examined consumption of nutrients instead of specific food group items. By assessing patterns of food group consumption, it may be easier to identify the cause of nutrient deficiencies [44] and design appropriate interventions. Studies assessing dietary behaviours may be limited by recall bias. Even provided with food descriptions, it is possible that students will not properly classify their consumption behaviours; this may be attributed to their difficulty conceptualizing portion sizes [45], or because portion sizes are often not provided in schools, snack bars, or restaurants. This may lead to erroneous estimates of student dietary behaviours since portions are most often overestimated [46]. The C_q_ questions using Canada’s Food Guide include a brief description of serving sizes and images. The use of food photographs helps in estimating portion sizes and increases the accuracy in estimation compared to unaided estimates [47,48], but only slightly [45]. Therefore responses to questions in the C_q_ may be more accurate depictions of youth eating behaviours.

There are several limitations to this study. Since this is the first time that these items have been used to measure dietary behaviours, there are no direct comparison studies. Second, the use of Canada’s Food Guide excludes any depiction in the measures of several food items which may be unhealthy (e.g., there are no specific depictions of oils or fats, or junk food/sugary beverage consumption). As well, youth consuming meals made of several ingredients may not be able to distinguish or divide meals into its constituent parts. Perhaps the addition of a ‘mixed foods’ category will better apportion the nutrients from mixed food products [49] while capturing consumption of certain unhealthy foods. Third, researchers relied on a small convenience sample of grade 9 students (age 14–15) from southwestern Ontario, which may have limited the generalizability of the findings. Fourth, while it would have been ideal to collect test-retest data for a longer period of time [24], the timeframe we used was consistent with other similar validation studies with youth populations in Canada [20,50]. However, there is little reason to believe that high school students in Ontario would respond differently to the survey than respondents in other jurisdictions. And fourth, it is possible that youth consuming foods/meals made up of several ingredients may not be able to distinguish and divide the constituent parts into the different food groups.

## Conclusion

Traditional measures of height, weight, and dietary behaviours are not always feasible for large-scale school-based studies. While the test-retest reliability of the measures used in this study were all high, the validity of the measures was considered acceptable. Despite few limitations, the results support the use of the C_q_ to obtain proxy measures of weight status and dietary behaviours in youth. This study is the first to contribute information on the use of the food guide for self-reported measures of dietary intake. The role of the four food groups in the Food Guide is well established in Canada, where Canada’s first Food Guide was introduced to the public in 1942 [51]. Comparing dietary behaviours to national Food Guide recommendations by using the recommendations themselves is a novel way to explore and understand the reach of the guidelines and youth dietary behaviours to help guide future interventions. In addition to identifying reliable and valid measures for future investigation of youth health behaviours, this study found that youth are not meeting dietary recommendations for achieving health benefits. With knowledge of the weight status and dietary behaviours of a representative sample of grade nine students, it is possible to guide program and policy development. Preliminary data from this validation study stress the urgency for additional policies and programs in schools to improve eating behaviours and reduce the risk of overweight and obesity among youth in Ontario; if eating behaviours remain as found in this study, youth susceptibility to overweight and obese will likely increase.

## Abbreviations

BMI: Body mass index; Cm: Centimeter; Cq: COMPASS questionnaire; CCHS: Canadian community health survey; CHMS: Canadian health measures survey; ICC: Intraclass correlation coefficients; Kg: Kilogram; kg/m2: Kilogram per meter squared; SR: Self-reported; T1: Time 1; T2: Time 2; WHO: World health organization; YRBS: Youth risk behavior survey; 1wk: One week.

## Competing interests

The authors declare that they have no competing interests.

## Authors’ contributions

SL developed the COMPASS questionnaire, was the principal investigator for the data collection for this study, conceived the manuscript idea, performed the analyses, interpreted the results, and contributed to the writing of the manuscript. RL contributed to the writing of the manuscript and the interpretation of the results. Both authors read and approved the final manuscript.
